# The Beginnings of Education

**Published:** 1915-08

**Authors:** Henry Jones Mulford

**Affiliations:** Buffalo, N. Y.


### BUFFALO MEDICAL JOURNAL

Yearly Volume 71. AUGUST, 1915. No. 1

### ORIGINAL ARTICLES

The right is reserved to decline papers not dealing with practical medical and surgical subjects, and such as might offend or fail to interest readers. Contributors are solely responsible for opinions, methods of expression and revision of proof.

### The Beginnings of Education.

Some Suggestions to Parents and Teachers.

By HENRY JONES MULFORD, M. D., Buffalo, N. Y.

First Paper—The Child.

The educational situation of today is somewhat "muddled;" but, if we wish to understand why it is muddled we have only to turn to the dictionary and look up the word "fad." A fad, we read there, is a matter of no importance, or an important matter imperfectly understood, taken up and urged with more zeal than sense. It seems to me that this definition is very "pat" to the situation at hand. Certainly many of the ideas now being entertained as educational may be assigned to the one or to the other side of the definition. In each direction the zeal for accomplishment has outstripped right judgment; the endeavor is well-meaning, but the endeavorer begins with a wrong assumption.

However, do not misunderstand me, I do not mean to say that all endeavor in this direction is upon wrong lines; all light does not issue from a will-o-the-wisp. There is some good work being done along educational lines, of course; but there is a more or less lack of rationality: the procedures have no real scientific basis. And that is what our system of education needs: an exact, a scientifically exact, basis from which to proceed. We gain little through the employment of hit-or-miss methods. The result is confusing to all concerned: to the teacher, and to the parent, to say nothing of the effect upon the child himself. But now, in seeking for the right procedure, in the working out of a scientific method, we do not need to seek very far: all we need to understand is the adjustment of the educational process to the facts of physical development. These facts embrace the entire organism, for, while the brain is the organ most concerned, all of the other organs have their part to play in the support of the brain. We shall need to understand how the human body has developed; we shall need especially to understand how and why the human brain has developed. Understanding these facts we shall know how to proceed in beginning, and in continuing, the education of the child.

I said a moment ago that all we need in education is a scientific basis from which to proceed; but, there is something else that must be mentioned in this connection. Not only must we adjust our system to conform to the individual, but, we must, also, fit the individual into the system. The human brain is very variable in regard to function, no two individuals of the same age are exactly alike in the exhibition of brain power. We must, therefore, find some method for determining the child's brain power so that we shall know where to place him in the educational scale. One fault of the present system is that from thirty to forty children are herded together in one room under one teacher. This system is unfair; unfair to the teacher and unfair to the children. The teacher does poor work because she has too much work to do, and the children, naturally, do not do their best under a teacher who cannot do her best. It may be said, also, that amongst forty children, there are some who are below grade, and some who are above. Those below are injured through the endeavor to endure beyond their strength; those above are retarded through receiving less than they can assimilate. What is needed is a system that will eliminate these irregularities. In these articles I shall offer some suggestions toward that end; but I am not deluding myself with the thought that my suggestions are going to solve all the problems of education. No one man may accomplish that. But, one man may make a beginning; and, if I have done that I shall be content.

The "facts of physical development" are found in the developmental history of the entire organism; but each organ has its own group of facts. The facts covering each organ give the history of that organ as an individual organ, and, as an organ associated with other organs. The organs of the human organism are an association of organs working together for the common good; they support one another, and they support the individual. Any organ failing to keep in line with the others sets up a disturbance that is reflected in the well-being of the individual. Such an individual immediately is thrown off the normal and he is unable to accomplish as much as he did when he was normal; both his physical and his mental strength are impaired. Such being the case, we readily can understand the importance of a complete general examination in an abnormal individual. And, in the case of a child, such an examination will possess its full value only when undertaken by a man who fully understands the evolutionary history of each organ.

But, now, while our investigation must include all the organs of the child's body, there is one organ which is at the head of all and to which we must give our chief attention. That organ is the brain. The adult brain holds all the secrets of man's development; but it is the child brain that holds the key that unlocks those secrets. We seek the developmental plan of the brain: how its cells are developed, and when the various centres come into action. The growth of the brain during the first eight years of its existence reveals that plan. Through study of the immature we learn the secrets of the mature.

We all appreciate, I know, that it is the grey matter of the brain that holds man's destiny; it is here that the "secrets" lie. This grey matter is found mostly upon the surface of the hemispheres, being not more than an eighth of an inch in depth. If this substance were just an even layer over the outer surfaces of the hemispheres there would be but very little of it, and man would not be the advanced creature he is; he still would be the chattering ape he was many, many thousands of years ago. The surface of the brain is convoluted, and we immediately see the importance of this arrangement. The development of the convolutions has given more brain surface to man, for now the grey matter dips down about every surface of these convolutions, and this has taken him out of the ape class. As man developed in the process of his evolution greater and greater demands were made upon his brain, and it was these demands that forced his brain to grow and the convolutions to form. But the brain could increase in size only with great difficulty owing to the bony walls closely surrounding it; it could develope grey matter through developing convolutions much more easily than through developing size, and, so, the ever-increasing grey cells made places for themselves by, as it were, carving out the convolutions. In fact, it is in the depth of the fissures that separate the convolutions and in the irregularity of the convolutions themselves that the importance of the man brain lies. In the brains of the lower monkeys there are almost no convolutions; in the higher apes the convolutions are well-marked, but they are large, coarse and uncomplicated. In prehistoric man the surface of the brain must have been but little more complex than that of the ape, for prehistoric man had little beyond the merely animal needs, and, so, his grey matter was enough only to meet those needs.

We have in the grey matter of the hemispheres the centres that control the external activities of the individual. These centres may be divided into groups. Group one would be made up of the centres governing the special senses; group two would be the motor centres, those governing muscular action; and group three would be the intellectual centres, those having to do with thought and reason. Now, these centres, while they are made up of the same kind of tissues, are not exactly alike. They differ in the size, shape and arrangement of their cells, and in the development of their activities. The centres do not all come into action at the same time, but follow a certain developmental sequence, the order being as follows: smell and taste, sight, hearing, tactile sensation, the motor centres, and, lastly, the higher centres.

There are two directions in which these centres must prepare themselves for functionating. First, the cortical cells must be ready for action; and, second, the fibres leading to and from the cells must be ready to transmit impulses. The cells develop first, and then the fibres; but the fibres when first formed are not yet fully capable of conducting impulses. Each fibre needs a protective covering, or insulating sheath, before it is quite ready for function. Investigation has shown that the afferent or sensory fibres, those carrying impulses to the centres, are the first to become invested with this sheath; and that the efferent or motor fibres, those carrying impulses from the centres, develop their sheaths next. Certain fibres which connect the various centres in the cortex, and which are called association fibres, are the last to receive their sheaths.

The fibres of the centres of special sensation are, mostly, afferent fibres, while the fibres of the centres in the motor area are both afferent and efferent. From this we can understand how these centres can receive impulses before they can respond to them. It is because their sensory (afferent) fibres are ready for work before the motor (efferent) fibres are. These areas are the first to functionate in the man brain. And, not only are these the first to functionate in the brain of today, but they were the first to functionate, the first to develop, in the prehistoric animal brain. They were the first animal centres, and they were the first man centres; they were the first cerebral centres needed by the animal organism. Now, as these were the first centres to devolop they must be the oldest, and I call them, therefore, the primitive centres. These centres have existed from, and before, the actual beginning of man; man always has used them and always will. It is obvious, then, that these centres must receive first attention in any scheme having to do with brain education.

The fact of great importance here is the dominance of the primitive centres; they dominate all the other centres because they are the oldest and the strongest. In the brain of the prehistoric animal these centres were the only centres in function; in the brain of prehistoric man these centres still dominated, but their action was becoming associated with the action of the new centres, the higher centres. The man is developing, but he is developing through the animal; it is the activity of the primitive centres that is developing the higher. This has gone on until the modern brain has been developed, but the modern brain is still the same kind of brain. It is a combination brain, a combination of animal brain and man brain, of primitive centres and higher centres. But in this combination the primitive area is still the dominant area, as it was in the primitive brain. The development of the higher centres still depends upon the activity of the primitive centres. I urge that this be not forgotten, for it is a point of some importance.

Up to this point we have given our attention to brain structure, but, in the study of the human brain, our interest lies in the direction of function as well as in the direction of structure. The two are inseparably connected. Structure comes first, of course, and function follows when structure is complete, function being the expression of the structure in action; but, perfect structure does not necessarily imply perfect function. While the capability for function is born with the brain that capability is not developed into complete function through mere increase in the brain mass. Function here is developed through external agencies, through action, through training.

The brain of the man of today is larger and heavier than the brain of the prehistoric man animal; but, so far as fundamental structure is concerned there is no difference. It is true that new centres have developed in the one, but the tissue in which these new centres are situated is the same kind of tissue as that in which the old centres developed. There has been an actual increase in the amount of cortex, but there has been no alteration in its fundamental character. The cellular elements differ in regard to shape, size, and arrangement in the two areas, but they remain the same cells so far as structure is concerned. We see, then, that, structurally, the animal brain and the man brain are the same. The man brain is no stronger structurally, but it possesses the high function of thought, which the animal brain does not.

This thought function is our greatest concern for it is that which gives man his position at the head of the animal scale. Without thought man becomes nothing more than a beast. But now ,there are two factors upon which the quality of thought depends: memory and fatigue. In fact, without memory there can be no thought; and, in the presence of fatigue, the thought process is more or less impaired. **It is memory that makes man; it is fatigue that unmakes him.**

It is in memory that the real strength of the brain lies, for it is memory that is the basis of all brain activity, especially of the higher centres in man. If man did not possess memory he could not remember; and, if he could not remember, he could not think, he could not reason, he could not achieve. He would be without ideas of any sort, for ideas cannot originate within a brain which cannot remember. In such a brain an idea can come, if it can come at all, only through an external stimulus: a sound, an image, a sensation, would arouse certain cells into activity, and an idea would be born as a result of the stimulus, but, the moment the stimulus was removed, the idea would vanish, for there would be no memory to hold it.

There are two kinds of memory: reflex and conscious. Reflex memory exists in all living cells; conscious memory is peculiar to certain cells. Reflex memory is memory that responds to an external stimulus; the cell knows how to act, but it does not act until told, it cannot direct its own action. The cells of the various organs of the human body have this memory. The organ cells perform the special function allotted to them when the proper stimulus comes to them. Conscious memory is memory under the control of the individual; the cells can revive the images stored in them. This form of memory exists only in the cells of the brain cortex. In fact, these brain cells have both forms of memory, the reflex and the conscious. They can respond to external stimuli and they can respond to stimuli originating within themselves; an individual can have images stored in his memory revived through some outside influence, or, he can sit down and revive them through pure brain effort. This is an important point in our understanding of the human brain; this double memory will explain a number of puzzling things in relation to brain action.

Is there a special centre for memory in the brain? I think we have answered that question above. If the cerebral cells possess conscious memory what is the need of a special centre? And, too, one centre could not take care of all the memory impulses coming into the brain; it would require a great amount of cortex, and would unnecessarily complicate an already complicated organ. I believe that each centre has its own memory, the vigor of the memory depending upon two factors: past experience of the centre, and the present physical condition of the cells within the centre. By past experience I mean training; and by present physical condition I mean any condition interfering with cell activity.

Fatigue is the condition interfering with cell activity that most concerns us here. Fatigue is our greatest enemy. It invades all parts of the organism, muscles, organs, everything, including the very brain itself. And the worst of it is that it comes through perfectly natural channels, that is, through the usual body activities. It is, only, in fatigue, that the action has become overaction; and overaction means overwork, and overwork means fatigue. Any organism suffering from fatigue is a tired organism, and a tired organism is a weak organism; it is one that breaks down easily. In this respect it may be said that fatigue is at the bottom of all disease; that it is the ultimate cause of disease. Now, man's real strength lies in action, action is the life of his muscles, of his organs; but, as it is his life, so it also may be his death. Normal activity encourages growth; overactivity retards growth. Action is the strength of man's organism, fatigue is the weakness of its strength.

Whenever a muscle contracts, or a brain centre acts, there occurs within the cells of the part concerned a chemical reaction. Now, a chemical reaction is a somewhat complicated process. It consists in the splitting up of the old compounds and the formation of new, with the liberation of force and heat. In the chemistry of living tissues the results of the reaction are of vast importance, for, upon their completeness, depends the well-being of the organism. In the living body the reaction within the cell may not be perceived, only the result becoming apparent. We know that heat and force have been produced, for we note the increase in the temperature of the part, and its increased capacity for action. Of the new substances formed within the cell some are of further use to the organism and are stored up, while the others are merely waste products. These waste matters are all harmful to the cell, some acting as mechanical obstructions, some as actual poisons to the cell substance. It is obvious, then, that, if the waste matters are allowed to remain within the cell, the cell cannot properly perform its function. It becomes necessary, therefore, to remove this waste from the cell as fast as it is formed if we want that cell to act properly. One of the functions of the blood is the removal of the waste. The plasma, the fluid portion of the blood, completely surrounds each cell and dissolves out from it these unneeded products of its activity. Later these products are removed from the blood itself through the action of the skin and kidneys.

If, now, these waste matters are formed faster than they can be removed the action of the cell becomes clogged or weakened; the individual suffers from fatigue in that part. An experiment by Dr. Alexis Carrell will illustrate this. Dr. Carrell cuts a small piece of tissue from a living muscle and keeps it alive outside of the body from which it was removed. He is able to do this by placing the tissue in a current of blood serum kept at the temperature of the body. This supplies the normal conditions under which the muscle grew in the body in all but one direction. All goes well for a time. Then the tissue begins to change; its activity becomes less and less, and, finally, if left there, it will die. But now, Dr. Carrell removes the piece of tissue from the blood serum, washes it in warm salt solution, and then replaces it in the serum. At once the tissue proceeds to take up nourishment from the surrounding fluid as it did before; it continues to live. What happened to that tissue before it was removed from the blood serum? It was being choked by its own waste. There being no vessels to carry off the waste products of the cell chemistry those products were retained to embarrass its action. The washing of the tissue in salt solution removed the waste and the cells were enabled to resume their former activity.

It is the same with a fatigued muscle or a tired brain. The fatigue originates through the crowding in upon the cells of the structure concerned of the products of its own activity; but the fatigue may be **removed** by washing from the part these products. This the blood does; and, once the fatigue-producing substances are removed, the cells return to their former vigor. But this return to normal may be modified by a too long period of the fatigue-producing activity ,or, by a too short period of rest following the fatigue. In either event there will follow some permanent alteration in the cell structure, and a permanent alteration cannot be changed back to normal.

In fatigue of the brain the cells of the grey matter are unable properly to perform their function because of the presence of this tissue waste. The cells lose their acuteness of perception and the incoming impulses are not received in the usual clearness. There is always an impairment of function, and, often, an entire suspension; the cell, under the influence of fatigue, is not itself. Now, if the function of the cortical cells is interfered with in this manner, it means, in its ultimate reaches, that it is the faculty of memory that is injured. For, if the cell cannot perform its function normally, if the sensory impulses are not received as they should be, if the receiving apparatus is weak, then the sensory impression is not going to be retained; the individual is going to forget. We can appreciate, then, just what fatigue does to man. The strength of his brain depends upon its thought faculty; the strength of the thought faculty depends upon memory; and, the vigor of the memory depends upon the presence or the absence of fatigue. As I said once before: It is fatigue that unmakes man.

As we have seen, the animal brain had no strength beyond the strength of structure: it had no thought strength. And the man brain had no thought strength until the thought areas became conscious, until the cells of the thought areas developed conscious memory. Although conscious memory came before thought, the development of thought and conscious memory went on together during the early epochs of man's existence. If, then, memory was coeval with thought and had its seat in the same cortical cells, that which affected thought would affect memory. If the higher centres were developed through action then must memory also have been developed through action. But the development of memory was slow, just as the development of the brain cortex was slow; both developed through infinite effort and over a long, long stretch of time. Even now the growth of memory is a matter of some pains. The brain cells are impressionable and respond quickly to sensory impulses, but they do not always retain the impressions brought to them; the ideas do not at once enter into the cell consciousness. Usually the same impulse has to be repeated over and over again in order to make the idea capable of being recalled; but, the vigor of the memory does not depend upon the number of times the same impulse is received by the cells. If it did teaching would be no task at all. That which does determine the strength of the memory is the manner in which the sensory impulse originates, and the condition of the cells receiving that impulse. In plain English, it is the method of the teacher, and the presence or the absence of fatigue.

The difference between the adult brain and the child brain is a difference of capacity. It is much the same difference that exists between the man brain and the animal brain. The child brain has the same structure that the adult brain has, but it hasn't the same development. The convolutions and the fissures are there, but the convolutions are not so complicated and the fissures are not so deep. The child brain is merely a child brain, and is no greater than the age of its possesser. That is, in the child under eight years of age, the brain is not yet in control. At this period of life the child is controlled by action; his brain is being developed through action, and is not yet strong enough to direct the activities of the individual. **In the child action controls the brain; in the adult the brain controls action.**

Knowing this we shall know how to approach the child brain; through it we learn that the brain of the child is the measure of the child's capacity. I mean by that that the child's capacity for study is determined by the age of his brain. Now, this does not mean, necessarily, the age of the child in years. It means the developmental age of the brain, the stage of development of the cortical centres. It often follows that the development of a brain is out of all proportion to the years of its possessor. We frequently see children of, say six years, who are as bright as those of eight, or, as dull as those of four; the brain development in the one being at the eight year period, and in the other at the four. It is obvious here that, if, in either case, we attempted to teach the child by the six year method we should be doing him an injustice In the first instance there would be no actual harm done; the result would be, only, that the child's progress would be retarded through failure to supply as much as the brain could take care of. But in the second the result would be vastly different. In this the matter urged upon the brain would be more than it could assimilate, and there would be actual harm done to the brain structure through this forcing of an immature organ. This harm might be such as permanently to cripple the action of the brain centres.

We should, then, be very careful as to how we approach the child brain. **I believe that no child should begin formal study whose brain is not eight years old.** My reason for that belief lies in the fact that the human brain does not complete its growth until about its eighth year. Before that time it is in process of growth, and, is not ready to assume the functions of the full-grown brain. In the early years of its existence the child brain is, in fact, no more than an animal brain; it is, almost wholly, a motor brain, a reflex brain as it were, a brain of action. It is a brain in the evolutionary stage; it is the prehistoric brain, bound by the limitations of its evolutionary ancestors.

The brain is at once the strongest and the weakest of the organs of man. It is the strongest because it is the organ in control; it is the weakest because it is the youngest of all his organs. In the process of the development of the major organs of the body the brain must have been the last for which man felt the need. It would be the youngest, therefore, because it was the last to develop. And, as it was the youngest in the evolutionary scale, so is it also, the youngest in the development of the child; as it was the last organ to develop during man's long, long, period of evolution, so is it now the last to be called into action after man is brought into this world. That is, the brain does not become a conscious instrument until some years after the birth of its possessor.

The growth of the brain in the child repeats the evolutionary history of that organ. The various epochs through which man has passed, each epoch having extended through many thousands of years, have left their marks upon the brain Among those epochs might be mentioned that of the brute-man whose strength and savage temper dominated everything; that of the cave-man whose beginning thought added crude cunning to the strength of the first; and that of the tribal savage into whose developing higher brain centres crept imagination and superstition. And, because each epoch endured through so long a period, the characteristics of each have left their indelible impress upon the brain cells; that which the brain centres did through so long a period then they do now through force of habit. The structure of the centres has been permanently marked, and we might, therefore, call these habits "structure-habits." These structure habits reveal themselves through the brain action of the individual. This will help to explain why some men seem out of all proportion to their environment: why some are all brute, some are all selfishness, and some mere star-gazing mystics.

The child brain attains its full growth at eight years; but, even then, it is not capable of full function. The higher centres are only just coming into activity. During the period of its growth it has been a motor brain, and it still is a motor brain, but now, the action of the old centres, the motor, is being complicated by the action of the new, the thought centres. The simple brain is becoming a complex brain. The complexity arises through the opening up of the higher centres; but we only have to remember that these higher centres came into being through the lower. The primitive centres were the dominant centres in the beginning, and they still are the dominant centres. The child brain being a primitive brain must develop as the primitive brain developed.

The primitive brain, then, the brain of action, is what most concerns us in the child; it is from this direction that the child may best be understood. The child is all primitive, all his impulses arise through the primitive centres. The "vagaries" of child life are seen to be just primitive impulses, impulses without thought behind them. There can be no real thought at this period for the thought centres are not yet in control. Thought began in man only after the motor areas had been developed; and the child, repeating within the period of a few years, man's long drawn out evolution, develops his thought as man developed his.

In conclusion I will remind you that there are two facts to be remembered here. First, that right brain action depends upon right development of the brain centres. Second, that the development of the centres comes through action. We cannot instruct a child whose brain centres have not developed; and, in order properly to instruct one whose centres have developed, we must know just when those centres are ready to receive instruction and how best to present it to them. In teaching there is but one direction through which sane results may be attained: we must remember that the primitive centres dominate. The higher must develop through the lower; the brain of thought comes through the brain of action. In short, the best way to teach a child, the best way to develop his brain is through his activities.

